# Extracellular Matrix-Derived Hydrogels as Biomaterial for Different Skeletal Muscle Tissue Replacements

**DOI:** 10.3390/ma13112483

**Published:** 2020-05-29

**Authors:** Daniele Boso, Edoardo Maghin, Eugenia Carraro, Mattia Giagante, Piero Pavan, Martina Piccoli

**Affiliations:** 1 Fondazione Istituto di Ricerca Pediatrica Città della Speranza, 35127 Padova, Italy; edoardo.maghin@phd.unipd.it (E.M.); eugenia.carraro@studenti.unipd.it (E.C.); mattiagiagante@gmail.com (M.G.); piero.pavan@unipd.it (P.P.); 2 Department of Industrial Engineering, University of Padova, 35131 Padova, Italy; 3 Department of Women and Children Health, University of Padova, 35128 Padova, Italy; 4 Department of Biomedical Sciences, University of Padova, 35131 Padova, Italy

**Keywords:** extracellular matrix, hydrogel, skeletal muscle, tissue engineering

## Abstract

Recently, skeletal muscle represents a complex and challenging tissue to be generated in vitro for tissue engineering purposes. Several attempts have been pursued to develop hydrogels with different formulations resembling in vitro the characteristics of skeletal muscle tissue in vivo. This review article describes how different types of cell-laden hydrogels recapitulate the multiple interactions occurring between extracellular matrix (ECM) and muscle cells. A special attention is focused on the biochemical cues that affect myocytes morphology, adhesion, proliferation, and phenotype maintenance, underlining the importance of topographical cues exerted on the hydrogels to guide cellular orientation and facilitate myogenic differentiation and maturation. Moreover, we highlight the crucial role of 3D printing and bioreactors as useful platforms to finely control spatial deposition of cells into ECM based hydrogels and provide the skeletal muscle native-like tissue microenvironment, respectively.

## 1. Introduction

Skeletal muscle (SKM) represents a tremendously complicated tissue to generate in vitro due to the complex and finely controlled interconnections occurring between its components, which finally form a mature tissue able to exert its contractile function. The complex architecture of SKM is characterized by a particular and well-described arrangement of muscle fibers and associated connective tissue [1]. The stabilizing element that maintains the complexity and contributes to ensure the tissue proper contractile function is the extracellular matrix (ECM) [2]. The ECM is comprised of a conglomerate of proteins of both structural and signaling characteristics alike. If from one hand the intricated layers of muscular ECM provide the physical structure for force transmission between contracting myofibers and their surrounding tissue environment [3], on the other hand the ECM acts as an embedding medium for essential supportive muscle components such as capillaries and motoneurons [4]. Moreover, muscle ECM houses a number of peptides (e.g., growth factors and cytokines) and cell types such as macrophages, fibroblasts, and the muscle-specific stem cells (called satellite cells, SCs) that reside in a quiescent state in a well-defined anatomic location, representing their niche [5,6].

Innovative tissue engineering approaches have been developed for years in order to dissect these complex mechanisms of interaction and to produce materials able to finely mimic the biophysical and biomechanical effects of muscle ECM on cells, and vice versa [7]. In the last years, lot of efforts have been pursued in vitro to obtain ECM-derived 3D scaffolds as microenvironmentally suitable products to home different cell types, for reproducing a functional SKM tissue and studying many processes related to SKM myogenesis in physiological and pathological conditions, rather than maturation or regeneration pathways of this unique tissue.

In this review, we introduced the SKM ECM in terms of protein composition, cellular components and molecular mechanisms as an essential starting point for describing different types of ECM-derived hydrogels until now developed for the study of cell-ECM interactions in SKM. A special attention is focused on the ability of the 3D constructs to recapitulate the biochemical cues that affect cell behavior at multiple levels. It is well-known that topographical cues applied to the hydrogels are fundamental for guiding cellular orientation and facilitating differentiation and maturation of the myogenic cellular component, as well as inducing the correct alignment of myofibers which finally develop a functional SKM [7]. For this reason, we summarized the most recent exploited approaches to induce different topographical characteristics to hydrogels, giving strength to the concept that a SKM-like scaffold not only should resemble the correct cell-ECM interactions but may also possess structural and biomechanical properties as similar as possible to the native SKM tissue. In this context, a finely controlled spatial cell deposition into the 3D scaffolds acquires peculiar importance in developing a SKM-like tissue. Herein, we give a detailed view of several applications of 3D bioprinting in SKM generation which allows to spatially control, in a precise manner, the deposition of biological material. Finally, it is mandatory to highlight the importance of SKM devices, namely bioreactors, to guide and monitor the maturation of such engineered muscle tissues [8].

## 2. Skeletal Muscle: ECM, Matrisome, Growth Factors, and Cellular Components

### 2.1. Skeletal Muscle Extracellular Matrix

The ECM is the acellular component of all tissues and organs, secreted by cells forming a dynamic network, which is constantly being remodeled in a tissue-specific manner [9]. In SKM, ECM accounts for 1–10% of the total muscle mass, coating the muscle fibers with a layer called the basement membrane, which in turn is made of two layers: an inner basal lamina, directly in contact with muscle fibers and an outer reticular lamina layer [10]. The ECM provides physical scaffolding for mechanical structure, orienting muscle fibers and bearing force transmission during myofibers contraction. ECM can also bind and store several soluble growth factors, which are released during ECM remodeling and degradation, creating a gradient of signaling molecules that are free to bind to the relative cell receptor and influence cell behaviors [11]. ECM actively participate in the biochemical cell signaling during muscle development and function, with ECM-cell interactions affecting gene expression and controlling most fundamental cellular behaviors such as proliferation, migration, polarity, differentiation, and 3D spatial arrangement [11]. Mandatory players of this complex game are integrins, specific receptors on the cell surface that are involved in converting the extracellular signals to intracellular response, allowing cells to sense their surrounding microenvironment and actively modulate their processes and behavior. Specifically, integrins link the actin microfilaments of the cytoskeleton with ECM, working also as a sensor of tensile strain [10]. ECM topography and elasticity can act as a source of stimuli for muscle fibers, serving as instructive cues for different cellular behaviors, mainly in the form of fibrous proteins with micro and nano-features [12].

#### Skeletal Muscle Matrisome

The SKM ECM is an extremely tidy mixture composed by a plethora of macromolecules, such as proteins, polysaccharides, and small molecules. It is mainly arranged of around 300 proteins, the so-called “matrisome”. In particular, two major classes of proteins have been classified: proteoglycans and fibrous proteins including collagen, fibronectin, laminins, and elastin. The proteoglycan class consists in glycoproteins covalently linked to glycosaminoglycan (GAG) chains. GAGs are anionic polysaccharides and include hyaluronic acid, keratan sulphate, chondroitin sulphate, and heparan sulphate domains. The highly negatively charged GAG chains allow water retention, conferring space-filling function, and growth factor binding. Examples include large proteoglycans, such as aggrecan and versican, and small proteoglycans, such as decorin [13]. The class of fibrous proteins comprises collagen as the most abundant component, which accounts for up to 30% of the total protein mass. Collagen has multiple fundamental roles such as to provide structural and tensile strength to SKM tissue, sustain cell adhesion and induce cell migration, and drive tissue development. Collagen is composed by three polypeptide α-chains that form a triple-stranded helix, which can assemble into macromolecule complexes, such as fibrils and networks. Although, a total of 28 different collagen molecules can coexist in a heterogeneous mix, generally a single or few types of collagen predominate in a tissue-specific manner, like collagen types I and III for SKM [13]. Fibronectin is another fibrous protein that plays an important role in cell adhesion and migration, being useful as substrate for scaffold coating and in vitro cell culture. Fibronectin binds the integrin adhesion receptors on cell surface, and it has been shown to be able to bind several growth factors, acting as reservoir for different molecules [14]. Another class of cross-linked glycoproteins is represented by laminins, which are mainly localized in the basement membrane of the SKM. They exist in many forms, depending on the different composition of chain peptides (e.g., α1, β1, and γ1). Laminins turnover during muscle embryogenesis, adult homeostasis and tissue regeneration are fundamental to control the precise and specific SCs activation, expansion and self-renewal [15]. Their main role is also exerted in the formation and maintenance of vascular structures, which is interestingly relevant in ECM scaffold fabrication for tissue regeneration [16]. A crucial protein in SKM ECM is elastin, whose tropoelastin monomers associate together to form elastic fibers, with the final aim to impart elasticity to tissue that undergoes repeated stretch cycles [9]. SKM continuously responds and adapts to environmental stimuli such as injury, trauma or overloading during strong exercises. Muscle activity is known to be a major regulator of SKM mass, with an increase or decrease in mechanical loading resulting in muscle hypertrophy or atrophy, respectively. Several studies have identified numerous signaling molecules involved in this activity-induced muscle growth, with insulin-like growth factor-protein kinase B/Akt-mammalian target of rapamycin (IGF-Pi3-Akt-mTOR) pathway as a positive regulator, and Myostatin-Smad3 as a negative regulator of muscle mass. Another important player is YAP (Yes-associated protein) that was identified to contribute to the regulation of muscle mass, as overexpression of YAP is sufficient to induce SKM hypertrophy and the amount of YAP protein is increased in SKM cells after mechanical overload. YAP has been demonstrated to be a crucial regulator of mechanotransduction with mechanical forces serving as inputs for the regulation of YAP, whose activity can be affected by ECM stiffness, substrate rigidity, or cell spreading [17].

### 2.2. Skeletal Muscle ECM Growth Factors

In healthy conditions, SKM formation is guaranteed from embryogenesis to adult life and a key role is played by several growth factors able to modulate multiple processes occurring in SKM tissue, such as maintaining the quiescence of SCs or modulating ECM-cells interactions. To recapitulate the SKM microenvironment in vitro, it is crucial to understand all the biochemical components characterizing the tissue in vivo. The specific response to growth factors can promote different cell processes, from proliferation, to migration and differentiation. It depends at least on the composition of the ECM, in particular to the presence of many Notch signaling molecules, adhesion molecules and proteoglycan, that are able to bind and modulate growth factors [18]. The signaling cascade starts with the production and secretion of soluble factors by cells and continues with the binding to specific transmembrane receptors on target cells. This process is finely regulated by different cell types, by the distance that growth factors can perform and by the type of receptors on target cells, but also by ECM degradation and interaction.

In this context, SKM ECM behaves as an indispensable reservoir of growth factors, which can be trapped into the ECM mesh, forming a gradient and acting as potent modulator of cell behaviors. The principal factors characterized in SKM tissue are transforming growth factor-beta (TGF-β), vascular endothelial cell growth factor (VEGF), platelet-derived growth factor (PDGF), fibroblast growth factor (FGF), hepatocyte growth factor (HGF), and insulin-like growth factor 1 (IGF-1) [9,19].

Specific roles are related to HGF, that is present in inactive form close to SCs and enhances cell activation upon injury, and similarly to FGF [20,21]. Together these molecules can stimulate cell proliferation supporting a significant increase in final cell amount. On the other hand, other factors such as IGF are necessary to increase cell differentiation and fusion [22]. The specific roles of the aforementioned SKM-related growth factors are reported on Table 1. In SKM tissue engineering approaches it is important to take into account the most effective combination of growth factors for cell culture media, as a fundamental step in vitro to support and control the first phase of cell proliferation and to properly induce muscle cell differentiation, in order to obtain functional and mature constructs [23].

### 2.3. Skeletal Muscle Cellular Components

Cellular components of SKM are fundamental mediators of the multiple signals provided by growth factors entrapped into the SKM ECM and, whilst they maintain the functionality of SKM tissue, they are dynamic players that can change their behaviors due to external stimuli. Each mature muscle fiber is a cylindrical multinucleate cell (less than 100 μm in diameter) innervated by a branch of motoneurons; the cytoplasm of each muscle fiber (sarcoplasm) contains myofibrils, the basic functional unit of SKM, which run parallels along the muscle axis [29,30].

Endothelial cells reside in SKM and provide an extensive array of growth factors and cytokines, which stimulate SCs and recruit macrophages during inflammation [31,32]. Although in the adult organism no significant endothelial cells turnover occurs, upon severe injury, angiogenesis process is essential for efficient regeneration. The ECM plays an important role in controlling new blood vessels growth from pre-existing ones. During angiogenesis, many ECM components are involved, such as hyaluronic acid, thrombospondin-1, glycoproteins and proteoglycans. For example, heparan sulphate proteoglycan domains are able to bind large amount of molecules including enzymes, ECM molecules and growth factors (e.g., angiogenic VEGF), affecting their bioactivity and half-life, thus modulating angiogenesis [33,34].

Each muscle fiber is innervated by one motoneuron, forming a motor unit through which the mechanism of excitation-contraction coupling occurs. Motoneurons generate electrical impulses, whose action potential propagates along the axon and trigger the release of acetylcholine in the synaptic cleft, known as the neuromuscular junction (NMJ). The postsynaptic membrane of the muscle fiber is then depolarized, converting the chemical signal into muscular contraction [29]. Different evidences have established the interactions between motoneurons and SKM ECM and, in particular, glycoproteins regulated the assembly of the postsynaptic apparatus of the NMJ, while β1 integrins have been shown to control cytoskeletal architecture and basement membrane structure in muscle fibers, which in turn play a critical role for NMJ organization and motoneuron axons growth [35]. The importance of the cross talk between neuromuscular components and muscle fibers was recently demonstrated also in vitro using a 3D bioprinted engineered muscle construct [36]. Collagen, fibrin and neurotrophic factors are involved in the reinnervation on injured SKM, showing how Schwann cells—ECM interactions are essential for nerve growth during peripheral nerve regeneration [37]. Glial Schwann cells are required for the function and maintenance of the NMJ, actively modulating the synaptic activity. Following an injury, they are essential for proper muscle reinnervation, guiding axonal regeneration, and restoring myoneural junctions [38].

In the in vivo setting, immune system exerts a crucial role in SKM homeostasis, in particular after damage with the involvement of complex cellular events aimed at recognizing the injured microenvironment and mediating muscle regeneration [39]. An SKM damage induces the invasion of the injury site by monocytes and macrophages, the principal inflammatory cells present in the injured muscles [40]. Two distinct phases of muscle regeneration can be distinguished: the first involves the expression of cytokines and the recruitment of pro-inflammatory M1-macrophages, in association with the activation of SCs; the second phase is driven by changes in cytokines production in a time-dependent manner and leads to the shift to the anti-inflammatory M2-macrophages. This switch is induced by interleukin 10 (IL-10) and TGF-β cytokines, AMPK activation and IGF-1 production, and impacts on ECM remodeling inducing the inhibition of SCs proliferation while promoting myoblasts differentiation [39].

This review does not present in detail the formation of muscle fibers, but it is important to highlight that, although in the postnatal stage and under normal circumstances, the number of muscle fibers remain constant, with very few fibers being replaced, upon physiological injuries or after muscle degeneration, adult regenerative myogenesis is observed [41]. Muscle regeneration relies on a population of adult resident stem cells, called SCs. They are normally in a quiescent state and reside in an anatomically microenvironment defined as the stem cell niche, which is juxtaposed between the sarcolemma and the basal lamina that surrounds the muscle fibers [42]. The niche acts as an instructive environment, where communication between ECM and SCs is critical in the balance between quiescence and activation and in maintaining the stem cell pool. SCs interact with microenvironment via physical and biochemical stimuli. They are able to bind ECM molecules such as collagen VI and fibronectin through integrin receptors and also to detect secreted paracrine factors by surrounding cells [10,15].

Fibroblasts, which reside in the interstitial space between muscle fibers, secrete the majority of SKM ECM, including molecules such as collagen I, III, VI, fibronectin, metalloproteases (MMPs), and proteoglycans. These cells are responsible for maintaining the healthy muscle structure, providing mechanical stability and functionality to the muscle fibers. Fibroblasts and SCs reciprocally cross-talk during muscle regeneration, with fibroblasts acting as a component of the niche and as paracrine growth factor source, positively regulating SCs expansion and differentiation timing, and ensuring an efficient and proper muscle regeneration process [43]. Murphy et al. demonstrated that in vivo ablation of fibroblasts resulted in premature SCs differentiation, smaller myofibers and incomplete muscle regeneration [44]. Urciuolo et al. demonstrated that fibroblasts were crucial to augment muscle stem cell repopulation of decellularized ECM scaffolds in vitro, especially promoting SCs migration [45]. Up to date, several cell types were used to generate SKM in vitro, from embryonic stem cells [46] to induced pluripotent stem cells (iPSC) [47], from fetal stromal cells [48] to adult ones [49]. However, the best cell source to reproduce SKM in vitro is still to be found. Several works demonstrate that a mixed population composed of different—if not all—SKM cell components are necessary to generate in vitro a complete and mature muscle substitute [45,50].

## 3. Cell-Laden Hydrogels in Skeletal Muscle

Multiple approaches have been developed during recent years to engineer SKM in vitro [51,52,53] and, to date, hydrogel fabrication represents an optimal opportunity to produce 3D scaffolds finely resembling the native SKM tissue composition and structure. As defined by Peppas, “hydrogels are hydrophilic, three-dimension networks, which are able to imbibe large amounts of water or biological fluids, and thus resemble, to a large extent, a biological tissue” [54]. The advantages of hydrogels in respect to others engineered products are: (i) tunability regarding structure, shape, and mechanical stability, (ii) feasibility to predetermine topographical cues, and (iii) functionalization with growth factors and other bioactive molecules [55,56,57]. Cell-laden hydrogels possess the ability to recapitulate the cellular environment of SKM, and the blending of different cell types is easily achievable due to the aptitude of hydrogels to be highly biocompatible. In this context, hydrogels’ composition acquires paramount importance if considering the numerous components constituting the SKM tissue as multiple cell types and ECM proteins, and the complexity of interconnections occurring between them. During last years, a multitude of cell-laden hydrogels, that can be divided in different classes based on their formulations, has been produced: (1) natural, (2) synthetic, (3) mixed, and (4) decellularized ECM (dECM)-based hydrogels [58], each with its own pros and cons (Table 2).

### 3.1. Natural Hydrogels

Natural hydrogels are primarily composed by natural materials (mostly ECM derivates) such as agarose, alginate, chitosan, collagen, gelatin, fibrin/fibrinogen, hyaluronic acid, and silk. The advantages of this type of hydrogels are the inherent bioactivity and the structural resemblance to tissue ECM [58]. In SKM tissue replacement approaches, natural hydrogels represent a favorite choice due to high efficacy in triggering the SKM regeneration process. Several examples of hydrogels derived from natural materials reveal the importance of mimicking the ECM composition for the maturation of SKM scaffolds. Collagen, the major component of the ECM, and gelatin in its denatured form, have been widely used in SKM tissue engineering.

Pollot et al. tested collagen, agarose, alginate, fibrin, and collagen–chitosan hydrogels for their tensile mechanical properties and ability to regenerate SKM in vitro. Once stretched, the hydrogels were able to activate SCs, since they elongated at least twofold without failing. Based on RNA expression of myogenic markers expressed by primary rat SCs seeded onto the constructs, they concluded that fibrin has the best potential as a scaffold for SKM regeneration, followed by collagen. They propose to implant these hydrogels in a volume muscle loss (VML) model in future applications [59]. Mathias, Neal, and Heher embedded myoblasts in fibrin hydrogels and tested the efficacy of the construct to induce myofibers maturation in a murine *tibialis anterior* muscle defect, to form mature myotubes, and to better elongate myotubes after applying a tension, respectively [60,61,62].

Collagen is a major ECM component and its denaturated formulation has been deeply investigated for SKM regeneration. Chen and Paguirigan produced 10% (*w/v*) gelatin solutions, 1% microbial transglutaminase and 0.02% (*w/v*) chloroform to create crosslinked hydrogels blended with C2C12 myoblasts, applying a 10% static tension for 7 days. They found out that the cellularized gelatin scaffolds had greater elongated cells with projections respect to acellular control samples [63,64].

Marcinczyk et al. investigated the addition of ECM protein laminin to fibrinogen scaffolds as a suitable microenvironment for myoblasts. Incorporation of laminin increased myoblasts proliferation and secretion of growth factors related to regeneration processes. Furthermore, the combination of tensile and electrical stimulation improved cellular alignment and triggered an increased secretion of growth factors, indicating the high potential of fibrin–laminin hydrogels in SKM regenerative medicine [65].

Despite the high biocompatibility offered by naturally-derived hydrogels, low viscosity represents a constrain to be overcome because limits their direct implantation and reconstruction of load-bearing tissues, as well as their utilization for 3D printing applications [66].

### 3.2. Synthetic Hydrogels

Synthetic hydrogels are composed by synthetic materials such as polyurethane (PU), polyethylene glycol (PEG), polylactic acid (PLA), and polyvinyl alcohol (PVA). Synthetic hydrogels permit but do not promote cellular function, yet there are many ways to tether bioactive cues into these hydrogels. In addition to be less bioactive in comparison to the natural-derived, their intrinsic hydrophobicity generally results in poor cell adhesion, which leads to suboptimal proliferation and differentiation, ultimately resulting in substandard tissue formation [67]. The advantage of synthetic hydrogels is the higher tunability of the mechanical properties and capability of increasing mechanical strength and viscosity in respect to natural hydrogels [68]. Pure synthetic hydrogels are a less popular choice for SKM tissue engineering compared with natural hydrogels. Xu et al. investigated the effect of several different mixed hydrogels composed by N-isopropilacrilammide (NIPAAm) and 2-hydroxyethyl methacrylate (HEMA) on the differentiation of rat bone marrow mesenchymal stem cells (MSCs) into muscle tissue. Comparing different stiffness, the highest differentiation amount occurred when hydrogels with a 20 kPa elastic expansion modulus were used [69]. Another well-characterized synthetic material for SKM hydrogel preparation is poly(ethylene glycol) dyacrilate (PEGDA). Vannozzi et al. set up a self-folding PEGDA system similar to a muscle fascicle which can deliver cells while allowing nutrient and oxygen exchanges [70].

In another study, Browe and colleagues combined PEGDA with acrylic acid (AA) to form a biocompatible, electroactive hydrogel to be used as actuator while promoting mature SKM tissue. C2C12 cells survived and were metabolically active on 100% PEGDA, 1:4, 1:8, 1:12, and 1:16 PEGDA to AA hydrogels over a 10-day period. They observed that the contractile strength of the hydrogels increased together with an increasing amount of AA, demonstrating that AA was necessary for actuation of the constructs. They produced a synthetic biocompatible scaffold, actuating hydrogel for SKM with a reversible and repeatable response [71].

### 3.3. Mixed Hydrogels

A useful approach to ideally combine the best attributes of both natural and synthetic polymers for SKM regeneration is represented by mixed hydrogels. They are composed by at least one synthetic and one natural polymer. Fuoco et al. blended swine muscle-derived pericytes into PEG-fibrinogen hydrogel and immediately added growth medium to support the encapsulated cells. They demonstrated an efficient myotube formation and, after 30 days from the subcutaneous implantation in the back of immunocompromised mice, with the presence of mature myofibers and blood vessels [52]. Several synthetic polymers have been also combined with the natural material alginate. Rich et al. mixed alginate methacrylate with PEGDA prior to freeze drying to make microporous hydrogels with average ice crystal diameters ranging from 20 to 60 µm. These hydrogels were blended with the arginylglycylaspartic acid (RGD) peptide to facilitate cell adhesion of loaded mouse myoblasts. This resulted in increased infiltration of myoblasts inside the matrix, improved cell viability and gained expression of muscle creatine kinase and myosin heavy chain proteins [72]. Hwang et al. developed a gelatin PEG tyramine hydrogel encapsulating basic FGF (bFGF) and human adipose-derived stem cells. The authors found that treatment of a torn mouse *gastrocnemius* muscle in vivo with the protein-hydrogel resulted in low fibrosis, regained muscle contractility, and increased regenerating myofibers after 4 weeks [73]. Mulyasasmita and colleagues utilized protein-PEG hydrogels to deliver human iPSC-derived endothelial cells and VEGF after an ischemic injury in the *gastrocnemius* muscle of non-obese diabetic severe combined immune deficient mice. After 2 weeks, the muscle tissue showed reduced necrosis, and high number of myofibers compared with delivery of the hydrogel alone or PBS injection as control [74].

### 3.4. Decellularized ECM-Based Hydrogels

**Figure 1:**
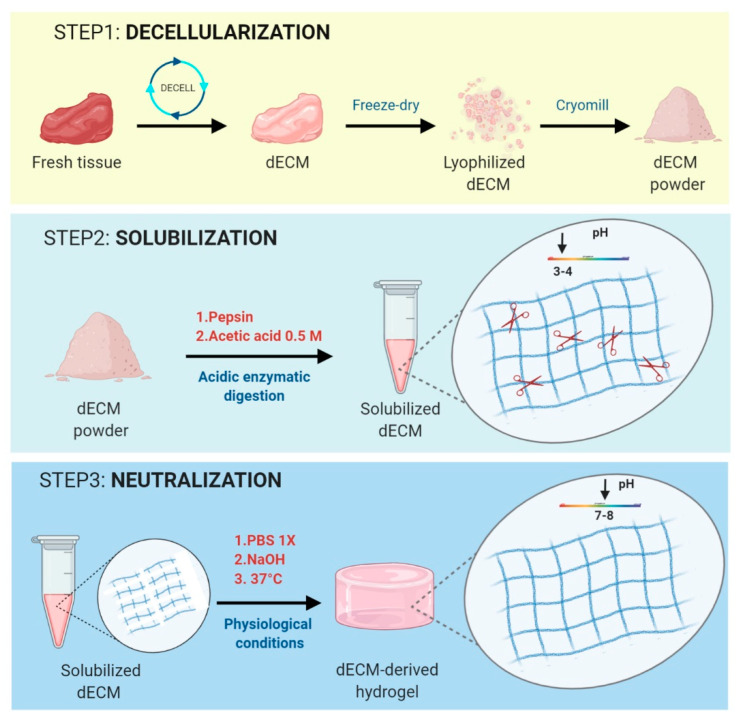
Production of decellularized extracellular matrix (dECM)-derived hydrogels via acidic pepsin mediated digestion. The method to produce dECM-derived hydrogels via pepsin-mediated digestion follows three steps. STEP 1: Decellularization of fresh tissues following a specific protocol for the tissue of interest (DECELL), followed by freeze-drying of dECM and cryomilling of lyophilized dECM to produce a fine dECM powder. STEP 2: Solubilization of dECM powder with pepsin to digest dECM collagen fibers under acidic conditions (pH 3–4). Red scissors: pepsin molecules. STEP 3: Neutralization of acidic conditions to physiological osmotic conditions (pH 7–8) for inducing the spontaneous reformation of intramolecular bonds between the digested collagen fragments into a homogeneous gel (dECM-derived hydrogel). Schematic was made using BioRender (https://app.biorender.com).

Due to the complexity of SKM ECM in terms of composition and topography, no natural, synthetic or mixed hydrogels can fully replicate all the features of native ECM [75]. The synthetic scaffold-based constructs partially fail to capture the detailed properties compared with their native counterparts in terms of structure, dynamics, biocompatibility, and function [75]. Decellularization method, defined as the process of removing resident cells from a tissue while preserving the ECM structure and components, such as laminin, collagens and GAGs, represents a useful technique to maintain as intact as possible the native tissue architecture [45,76]. The achieved dECM is a microenvironment devoid of cells that facilitates reseeded cell-ECM connections, and 3D cellular organization similar to those of living tissues. These dECM materials can be utilized in a variety of ways [77]: (i) as 3D scaffolds for recellularization approaches, (ii) as acellular patch-type, (iii) as injectable gel-type, and (iv) as feeder-layer for 2D cell culture. Indeed, one of the most important advantages in the use of dECM is the opportunity to fabricate different formulations supporting several needs and experimental or clinical questions. In this context, a paradigm is that a cell-specific dECM is required to maintain the cellular functions and phenotypes, due to the presence of peculiar growth and differentiation factors held in the dECM [75]. SKM-derived dECM bioscaffolds can be transformed into hydrogels, exploiting a collagen-based self-assembly process that is regulated in part by the presence of GAGs, proteoglycans and other ECM proteins [78]. The formation of a dECM-derived hydrogel consists in three fundamental steps: (i) decellularization of tissue to obtain a proper dECM, (ii) solubilization of dECM material into protein monomeric components and, (iii) temperature and/or pH controlled neutralization to trigger spontaneous reformation of intramolecular bonds of the monomeric components into a homogeneous gel (Figure 1). One of the most utilized methods to induce the formation of an ECM hydrogel is via pepsin mediated solubilization of a minced powder form of dECM [79]. Pepsin cleaves the telopeptide bonds of the collagen triple helix structure to unravel collagen fibril aggregates [80]. Gel formation starts from the neutralization of the liquid solubilized ECM to physiologic pH, salt concentration (ECM pre-gel) and temperature in vitro (ECM hydrogel) in an entropy driven process dominated by collagen kinetics. Several authors exploited this “gelation” method for producing SKM dECM-derived hydrogels [81,82,83,84].

A recent study by Perez and Ahearne investigated the impact of decellularization protocols in ECM-derived hydrogels obtained from porcine corneas. They isolated porcine corneas and decellularized with SDS, Triton X-100 or freeze-thaw cycles and demonstrated as diverse decellularization protocols had a different impact on the final characteristics of ECM-derived hydrogels [85]. As for porcine connective tissues, Fu et al. similarly studied the impact of different protocols of decellularization on the formation of ECM-derived hydrogels in porcine SKM tissues. Five multi-step methods were used to decellularize the SKM via rinsing in SDS, trypsin, ethylenediaminetetraacetic acid (EDTA), Triton X-100 and/or sodium deoxycholate at 4–37 °C. Hydrogel formation was induced via pepsin digestion and neutralization of pH to physiological conditions. They observed that dECM produced within sodium deoxycholate and Triton X-100 treatment formed a three-dimensional hydrogel scaffold material, while the matrix produced by SDS treatment was unable to form hydrogels, suggesting that SDS treatment was more aggressive and induced a stronger damage to the ECM proteins [82].

These results highlight the importance of finding the optimal decellularization method for the development and production of ECM-derived hydrogels and how different tissue sources require a specific method to obtain a dECM suitable for the formation of hydrogel scaffolds.

**Table 2:** Different types of hydrogels utilized for skeletal muscle (SKM) tissue engineering approaches. Corresponding references are cited for each type of hydrogel. Advantages and disadvantages are summarized for each category.

Type	Composition	Advantages	Disadvantages
Natural	Agarose, alginate, chitosan, collagen, gelatin, fibrin/fibrinogen, hyaluronic acid, silk [59,60,61,62,63,64,65]	High bioactivity, structural resemblance to tissue ECM, high efficacy in triggering the SKM regeneration process	Low viscosity, not suitable for direct implantation and reconstruction of load-bearing tissues, limit for 3D printing applications
Synthetic	N-isopropilacrilammide (NIPAAm)-2-hydroxyethyl methacrylate (HEMA), PEGDA, PEGDA-acrylic acid (AA) [69,70,71]	High tunability of mechanical properties such as increasing mechanical strength and viscosity	Low bioactivity, poor cell adhesion, suboptimal proliferation and differentiation, substandard tissue formation
Mixed	PEG-fibrinogen, alginate methacrylate-PEGDA-RGD, gelatin PEG tyramin, protein-PEG [52,72,73,74]	Combination of natural and synthetic	Combination of natural and synthetic
Decellularized ECM-based	Decellularized ECM from different tissues [82,86,87]	High bioactivity, native-like tissue environment, high maintenance of growth and differentiation factors, high tunability	Poor mechanical properties, fast degradation rate, low efficacy in loading, encapsulating and controlled delivery of cells or drugs

## 4. Biochemical Cues in Skeletal Muscle Tissue Engineering Approaches

One of the most important goals of tissue engineering is to mimic the physiology and functionality of native tissues. In this scenario, the strategies for SKM tissue engineering comprises many aspects including the improvement of muscle cells alignment and the involvement of other cell types to enhance innervation and vascularization [88]. In accordance with the idea that biomaterials should respect the following criteria: (i) to gradually biodegrade proceeding in parallel with the regeneration, (ii) to possess and induce the correct spatial organization, and (iii) to be reproducible, it is necessary to focus on the inclusion of growth factors in tissue engineering constructs, since their incorporation is required to optimize the scaffolds repopulation, but also to increase the regenerative capacity of the constructs once implanted in vivo [89]. Given that growth factors are soluble, in the last decades an increasing effort was spent on growth factors’ immobilization to control their maintenance and release, and to counteract their rapid degradation and loss of bioactivity. Immortalization approaches comprise noncovalent, i.e. encapsulation, adsorption, ion complexation, or covalent bindings, depending on properties of both growth factors and substrates [90]. The above-mentioned strategies, when applied without altering growth factors’ properties, provide the surveillance of peptides’ release. In order to enhance muscle cell proliferation and differentiation, many scientists have recently translated the results obtained with standard growth factor enriched media to 3D constructs.

Grasman and colleagues, for example, demonstrated how the absorption of HGF and its time-dependent release in fibrin scaffold, could enhance C2C12 growth [91].

Hwang et al. combined the use of human adipose derived stem cells and bFGF incorporated into gelatin-PEG-tyramine (GPT) hydrogels to enhance muscle regeneration in a VML mouse model, that resulted in decreased fibrosis, functional recovery, revascularization and reinnervation [73].

Liu et al. prepared alginate microbeads containing growth factors, including VEGF, and mixed urine-derived stem cells and collagen I gel with the microbeads. They used these formulations for in vivo studies through subcutaneous injection into nude mice and demonstrated that the combination of growth factors released locally from the alginate microbeads induced urine-derived stem cells to differentiate into a myogenic lineage, enhanced revascularization and reinnervation, and stimulated resident cell growth in vivo [92].

Ansari and colleagues set up a complex injectable alginate scaffold including bFGF to deliver gingival MSCs in order to promote differentiation into SKM upon in vivo implantation, paving the way for other numerous applications in muscle tissue regeneration [93].

### 4.1. Cell Activation for Growth Factors Production

An innovative field has emerged in the recent past to overcome the limitations associated to the in-situ delivery of growth factors, in particular for the enhancement of bioactivity and reduction of high cost and host-mediated enzymatic inactivation of the exogenous growth factors generally used [94]. These strategies consist in the modification of target cells to produce high concentration of specific factors. Two major approaches can be used to transfer safely the genetic material to the targeted cells: viral and non-viral vectors. Both these techniques manifest pros and cons: the viral strategy has higher efficiency, but also potentially high immune response (Adenoviruses mediated transient transfection with high transduction efficiency, but with high immunogenicity; Lentiviruses mediated stable transfection with moderate transduction efficiency and low immuonogenicity), while non-viral vectors, as liposomes or synthetic vehicle particles exhibit less immunogenicity, but at the same time a reduced efficiency [89].

### 4.2. Hydrogel Embedded Growth Factor Release

A challenging hurdle to overcome in tissue engineering hydrogel-based strategies is the control of embedded growth factor release. The capability to protect enveloped molecules from degradation is an intrinsic property of hydrogels that can be modulated by controlling the host-mediated reabsorption. Efforts have been pursued to understand the hydrogel-based growth factor delivery system within theoretical modeling of the growth factors’ release profiles. Wang et al. studied the effect of geometry and spatial disposition of growth factors-laden hydrogels on release kinetics. They incorporated heparin into a hydrogel to provide retention for growth factors and demonstrated that the geometry of 3D-printed hydrogels could be modified in order to release growth factors with predictable kinetics over long periods of time [95].

Woojin et al. engineered a synthetic hydrogel to facilitate the co-delivery of pro-myogenic factors, such as Wnt7a, and MuSCs to skeletal muscles affected by severe trauma or muscular dystrophies. Interestingly, Wnt7a release rate could be controlled by modulating the polymer density of the hydrogel, and this release rate could be further accelerated through the proteolytic degradation of the hydrogel [96]. Recently, Huang et al. described an easy loading protocol with vacuum system for embedding growth factors into a dECM-derived scaffold. They developed a cardiac patch composed of a myocardial dECM scaffold and synthetic cardiac stromal cells (synCSCs) generated by encapsulating secreted growth actors from isolated human cardiac stromal cells. The artificial scaffold maintained its potency after long-term cryopreservation and supported cardiac recovery in a rat model by reducing scarring, promoting angiomyogenesis, and boosting cardiac function [97].

A special attention concerns the gene therapy performed using the scaffolds as bioactive mediators of growth factors’ release, as Stilhano et al. demonstrated with alginate hydrogels able to modulate growth factors release during time, in accordance with the variation in molecular weight of alginate [98].

## 5. Topography in Skeletal Muscle Tissue Engineering

### 5.1. Topographical Cues

To successfully reproduce a 3D scaffold able to mimic SKM tissue in vitro, the structural guidance for muscle cells should be provided to induce efficient differentiation. Engineering the topography of biomaterial substrates to determine cell fate takes advantage of the natural contact-mediated signaling events that occur between cells and ECM [99]. Various strategies have been developed to guide muscle cells such as ECM mimetic topographical structure and mechanical or electrical stimulation methods [100,101]. Example of topographic cues that influence cell morphology and organization include microscale topographical features presented by micropatterned substrates, aligned polymeric fibrous matrices mimicking native ECM proteins, and 3D scaffolds with anisotropic porosity within which myoblasts can organize into wide and long myotubes.

For fabricating aligned micro/nanostructures, numerous methods have been employed to obtain appropriate topographical cues, which are an easy way to achieve the formation of myotubes owing to the efficient interactions among cells. The advantage of this approach is the readiness and reproducibility of the supports, and the possibility to generate different shapes and geometries. Recently, Kim et al. described an innovative 3D printing method with a SKM-specific dECM to bioengineer biochemically and topographically mimicked SKM constructs. They combined a dECM methacrylate (MA) derived from porcine SKM (as a bioink) with fibrillated PVA to fabricate a uniaxially oriented dECM-MA patterned structure. They demonstrated that the printed and topographically predetermined constructs accelerated the myogenic differentiation of murine myoblasts in comparison to a simpler gelatin methacrylate (GelMa)-based cell-laden structure [87].

In another study, Jiwiawat et al. demonstrated the efficacy of 2D micropatterned structures in inducing human iPSC-derived myogenic progenitors to form highly aligned and contracting myotubes. Clear nuclear alignment of differentiated cells was observed and, depending on the width of micropatterned lines, different elongated myotubes were formed. Thus, topographical cues from micropatterning and physiological substrate stiffness improved the formation of well-aligned and multi-nucleated myotubes similar to myofibers, with spontaneous contractile behavior across the long axis of the pattern [102]. Recently, a new and simple method has been developed by Yang et al. to induce myoblasts alignment. They set up a modified plasma treatment on a hybrid polycaprolactone (PCL) scaffold consisting of melt-printed perpendicular PCL struts and an electrospun PCL fibrous mat. For the hybrid scaffold production, the surface of the electrospun mat was selectively roughened with a plasma process supplemented with a template. This innovative type of plasma-treated hybrid scaffold demonstrated strong potential as a biomaterial for muscle tissue regeneration because of the significant enhanced cell alignment in comparison with the use of a hybrid scaffold with a non-roughened electrospun fiber surface or a hybrid scaffold with the whole surface roughened [103].

Another appealing approach to characterize the contribution of different tissue-specific topographies in driving instructive cell niches (made of ECM) is represented by Silica BIoReplication (SBR). SBR is a process that converts biological samples into silica, faithfully preserving the original topography at the nanoscale. Tang et al. exploited this method to firstly demonstrate that the precisely replicated tissue topography harbored sufficient information to direct the fate of human MSCs without the need of exogenous factors such as soluble growth factors or immobilized ECM molecules. They suggested as the tissue microenvironment captured by SBR profoundly affected MSC biology, and that the topographical cues were sufficient in initiating and directing differentiation of MSC, despite the absence of any biochemical cues [104].

### 5.2. Stiffness and Elastic Modulus

Although topographical cues represent a fundamental element to be considered, mechanical properties of materials utilized for SKM tissue engineering approaches exert a significant influence on cellular behavior and consequently on tissue functionality. Mechanical properties relate to forces exerted on the material and the resulting changes in shape. Among them, stiffness plays a fundamental role when considering mechanotransduction processes, and it should match that of native SKM tissue allowing for cell exposure to relevant mechanical forces thereby influencing cell fate [105]. Mechanotransduction occurs via intracellular signaling, influencing cellular responses such as proliferation, differentiation, metabolic activities, and maintaining the tissue identity with regard to functionality [106]. In SKM tissue, substrate stiffness orchestrates the formation of functional myotubes and a finely engineered control over it can be achieved by changing variables such as the composition of multi-polymer composites, the molecular weights of polymer constituents, crosslinking agents and crosslinking times [105]. Gilbert et al. demonstrated that biophysical properties such as matrix stiffness and elastic modulus play crucial roles in muscle stem cells (MuSCs) self-renewal and function in muscle regeneration. They obtained a tunable PEG hydrogel platform by altering the percentage of PEG polymer in solution and produced hydrogels with a range of stiffness including a formulation that mimicked the elastic modulus of adult murine SKM. They demonstrated as MuSCs cultured on the tissue-specific stiffness hydrogel were able to self-renew and to generate stem cell progeny that could potently repair damaged muscle. These results laid a milestone for the future SKM tissue engineering approaches by providing insight into the potency of tissue stiffness on stem cell fate regulation [107].

The natural response of all materials to stress is not purely elastic but also has a viscous component such as occurs with living tissues. The viscoelastic response of a material is sometimes described through the dynamic or complex modulus, which is represented by storage (E’) and loss (E’’) of moduli. Similarly, for deformations resulting from shear forces, the shear storage modulus (G’) and the shear loss modulus (G’’) are frequently evaluated by rheology and oscillatory experiments [108].

### 5.3. 3D Printing in Skeletal Muscle Engineering

The recently developed 3D printing technology is an additive manufacturing method that promises to bridge the difference between artificially engineered and native tissues. Such 3D printing is emerging as a scaffold fabrication approach finely mimicking native tissue complexity [109]. Indeed, 3D printing is a tool to assemble scaffolds with a high precision and accuracy, creating intricately detailed biomimetic 3D structures [110]. Scaffolds generated by 3D printing can have complex micro-geometries and, in practice, a layer-by-layer stratification can precisely deliver different cells or mechanical cues in the designed 3D architecture resembling the tissue of interest. Printability is a fundamental element to be considered exploiting a biomaterial for scaffold production and parameters to evaluate the resolution of the 3D bioprinted components of the scaffolds become crucial [111].

SKM complexity can be dissected using this innovative technology and, nowadays, lot of efforts have been pursued to develop optimal biomaterials suitable for 3D printing.

### 5.4. Bioinks

The most important element in designing a successful 3D bioprinting approach is the bioink, which is defined by Groll et al. as a formulation of cells suitable for processing by an automated biofabrication technology that may also contain biologically active components and biomaterials [112]. The bioink represents the building block of bioprinted constructs with a crucial role to support and provide an appropriate environment for incorporated cells [113]. Different characteristics should be considered when developing an optimal bioink for 3D bioprinting application such as printability including viscosity, gelation kinetics, filament stability and biocompatibility, mechanical properties, and nutrient diffusion capacity. An accurate process is required for adjusting these multiple parameters in order to design a bioink satisfying the biofabrication window requirements, in order to obtain bioinks with suboptimal, yet passable, print fidelity, while maintaining cell viability. Since the native ECM varies from tissue to tissue, bioinks should properly mimic the ECM of the target tissue, to support proliferation and differentiation of the specific cell populations homing that tissue [114,115]. A bioactive bioink should properly facilitate cell-matrix interactions to allow remodeling processes and new ECM synthesis [116].

Additionally, bioinks should have the cells homogeneously distributed in suspension to avoid cell aggregation and deposition and to extend the bioprinting time for making larger constructs [117,118].

### 5.5. dECM-Derived Bioinks in SKM

As above mentioned, dECM scaffolds are tissue specific and, since they are extracted from the tissue itself, they are biomimetic and contain a variety of proteins, proteoglycans, and cytokines that can aid cells in precise differentiation, maturation and tissue formation [75]. dECM-based bioinks have been produced from different tissues, including SKM, and in most of the cases have been used to regenerate the same tissue of origin. It should be noted that, in addition to the choice of a specific tissue, it is possible to obtain dECM bioinks from subjects of different ages or diseases, greatly expanding the spectrum of applications. Indeed, not only the tissue specific composition, but also age, injuries, diseases or degeneration, such as fibrosis or chronic inflammation, are important conditions that change dECM features and influences on target cells. Although literature is scarce about dECM-based bioinks for SKM, some examples highlight the potentiality of such approach. 

Choi et al. developed functional muscular constructs using a porcine SKM-derived dECM bioink, containing C2C12 cells and printed different patterns. They cultured the constructs for 7 days and observed high cell viability and increased cell proliferation compared with those prepared with collagen only. Moreover, high myogenic gene expression of C2C12 encapsulated into the dECM was observed at day 14 after differentiation induction, together with the preservation of ECM components and agrin, which allows the prepatterning of acetylcholine receptors [81]. More recently, the same group developed a novel VML treatment based on a 3D cell printing and SKM dECM-derived bioink that ensured both an organized structure and cell delivery with high viability [86]. Kim et al. employed a porcine SKM-derived dECM as biochemical component and a modified 3D cell-printing process to produce an in situ uniaxially aligned/micro-topographical structure. Myoblasts laden in the printed structure were aligned and differentiated with a high degree of myotube formation, owing to the synergistic effect of the SKM-specific biochemical and topographical cues [87].

## 6. Bioreactor Platforms in SKM Tissue Engineering Approaches

One more key element that needs to be addressed for SKM tissue engineering is choosing proper stimulation strategies (either mechanical-, electrical-, or electromechanical-stimulation) in order to obtain mature and functional biocompatible substitutes. Over recent years, multiple approaches have been developed to design suitable platforms ensuring the abovementioned stimulation strategies, and in particular, the use of bioreactor systems. Bioreactor is defined as an in vitro culture system that has been designed to mimic the physiological conditions of the tissue of interest, acting on cell behavior, metabolism, and interaction within constructs in order to achieve the proper tissue structure, organization, and ultimately maturation and function [119]. In this context, several types of bioreactors have been developed and manufactured to mimic the in vivo SKM tissue environment physiology, providing a powerful and reliable model to study cell-ECM interaction during myogenesis and regeneration. Furthermore, engineered SKM should be a reliable pre-clinical platform useful to accurately predict drug efficacy and toxicity in SKM-related human diseases [120].

The appropriate bioreactor settings should be developed in relation with the type of biomaterial used for bioscaffold production and taking into account the cues that encapsulated cells require to produce mature tissue. Myogenic cells highly respond to their microenvironment such as the surrounding ECM, mechanical forces, and biochemical signals [121]. Adequate physiochemical and structural properties as well as bioactive cues like incorporated growth factors to enhance cell adhesion, distribution, and myogenic differentiation can be added to the bioreactor platform finely mimicking the SKM microenvironment observed in vivo. The application of peculiar stimulation strategies has been pursued by researchers to recapitulate and trigger myocytes alignment, fusion and differentiation. Until now, two main strategies of stimulation have been adopted: the mechanical and electrical stimulations, and their combination [121].

The suitable mechanical environment can be simulated by cyclic and/or static strain, whereas the neural inputs are mimicked by electrical pulses. Kim et al showed that the relationship between mechanical and electrical stimulations were crucial to obtain in vitro the proper maturation of mature myotubes and organized tissues [122].

### 6.1. Mechanical Stimulation

Mechanical stimulation represents a fundamental element for myogenic cells and plays a critical role in the regulation of SKM mass, cell differentiation, tissue ECM remodeling and homeostasis. In several studies it has been shown that a specific type of stimulation (continuous versus stretched/relaxed stimulation) affected cells distribution, organization but also metabolism, protein synthesis and maturation [123,124,125]. The strategy and type of mechanical stimulation applied to the constructs triggered different adaptive response and consequently maturation of the tissue. The main aspect that mechanical stimulation aimed to replicate is the progressive (continuous stimulation) bone elongation, which occurs during muscle embryonic development and is crucial to organize the structure directionality of the tissue, together with the muscle cyclic elongation that occurs during exercise and homeostasis in adulthood (cyclic stimulation). For instance, bone growth elongation can be simulated using a ramp of continuous stretching that is essential to define myotube organization, whereas cyclic stimulation is crucial for the regulation of metabolic activity and growth, regulating glucose uptake and lactate efflux [125]. Moon et al. indeed showed the importance of bioreactor preconditioning in vitro to accelerate SKM tissue organization, maturation, and contractility of an engineered construct before in vivo application. They observed enhanced functionality of the tissue engineered construct after one week of bioreactor preconditioning culture (using cyclic stimulation) [126].

The application of combined stretch protocols has produced controversial outcomes with both promising beneficial and negative effects. The different observations are likely due to the amount of stretch, frequency and loading protocols applied during the culture into a specific bioreactor setting. Nevertheless, combined ramp and cyclic stretching protocols are essential to fully replicate the transition from an embryonic to a mature mechanical environment in which the muscle is continuously stimulated. Moreover, mechanical stimulation enhances the cross-striations of myotubes and a switch of myosin heavy chain (MYHC) isoforms from embryonic to adult. An appropriate mechanical load protocol can modulate and exert positive effects on gene regulation and protein expression, for both proliferation and differentiation of engrafted cells [106,127,128].

### 6.2. Electrical Stimulation

Another essential element for muscle maturation in vitro is represented by electrical stimulation recapitulating nerve pulse occurring in vivo. As observed for the mechanical load, the protocols integrated into bioreactors can differ in electrical field, pulse width, impulse frequency, and work:rest ratio. The electrical stimulation protocols need an accurate design before the application, because high electrical fields can induce membrane damage, decrease force generation during functional testing, and prevent force increase [129].

Studies on 2D monolayer cultured myotubes were performed to model the electrical stimulation in vitro and different patterns of impulse and stimulation were investigated in order to evoke fast versus slow contracting myofibers formation. When this kind of stimulation was translated to an engineered 3D construct a key point was what type of stimulation should be applied and the proper time during the culture in order to obtain the desired tissue maturation [130].

The different types of electrical stimulation modulate cytoskeletal rearrangement, at the beginning, and gene and protein expression, subsequentially. Khodabukus and Baar showed the intrinsic relationship of MYHC expression and chronic electric stimulation upon 14 days of culture. They set up a protocol altering contraction duration (0.6, 6, 60, and 600 s), while using a constant pulse frequency of 10 Hz. They demonstrated reduction in fast MYHC content with longer contraction duration and observed that all regimes induced an oxidative fiber type phenotype. As evidenced, this aspect is mandatory in order to obtain and model the correct engineered tissue construct that resembles specific muscle phenotype [131].

## 7. Hydrogels-Based Models of Skeletal Muscle-Related Diseases

In vitro human SKM disease models represent nowadays a potential way to overcome the failure in translating results gained from in vivo drug testing to the clinic. They can more closely mimic human pathologies conditions concerning tissue and organ specific cell types using patient-derived cells reflecting the patient’s individual SKM physiology and the disease progression in the myopathic state [132,133]. In this context, some studies have focused explicitly on designing hydrogel scaffolds and cells that both mimic the muscle pathophysiology and can be potential platforms to screen experimental drugs for SKM diseases. Among the SKM myopathies, a lot of efforts have been concentrated on developing the abovementioned systems for muscular dystrophies.

Maffioletti et al. showed the generation of 3D engineered SKM from human iPSCs derived from Duchenne (DMD), limb-gridle and muscular dystrophies patients. They induced 3D skeletal myogenic differentiation of iPSCs using hydrogels under tension to provide myofibers alignment. Artificial muscles recapitulated characteristics of human SKM tissue and pathological hallmarks of incurable forms of severe muscular dystrophies could be modeled with high fidelity [53].

Serena et al. reported the in vitro fabrication of human myotubes carrying genetic diseases, based on micropatterned, polyacrylamide hydrogels coated with either laminin, fibronectin, or Matrigel. Human myoblasts derived from biopsies of both healthy and DMD-affected donors were cultured onto micropatterned polyacrylamide hydrogels, and later showed increased alignment, differentiation and sarcomeric striations depending on hydrogel stiffness and the coating protein. Furthermore, myotubes cultured on polyacrylamide hydrogels were endowed with the ability to recapitulate particular pathological hallmarks, such as the decreased expression of dystrophin in DMD (compared with healthy myotubes) and the unchanged production of sarcomeric units, which is known to be independent from dystrophin expression in the case of DMD [134].

Vandenburgh et al. used dystrophic myoblasts from *mdx* mice that were incorporated in natural hydrogels (collagen type I or fibrin) that were cast around posts. The resultant myotubes were electrically stimulated and contractile force generation was measured. In addition, 31 compounds that have the potential to serve as DMD drugs were screened by measuring changes in force generation upon treatment. Among the 31 compounds tested, 11 significantly increased the tetanic force when compared with placebo-treated controls. Interestingly, they were able to recognize beneficial compound interactions in addition to the deleterious interactions of combinatorial compounds routinely administered to some DMD patients [135]. The similarity in response of in vitro *mdx* muscle models and DMD patients to many identical compounds clearly demonstrates the potential of this system as a useful pre-clinical model [136]. Despite very few data are present in literature about the use of diseased ECM from SKM to produce hydrogel scaffolds resembling affected environments, intriguing studies should be performed in the next future to deeply analyze the role of pathological ECM on tissue resident cells, experimentally crossing healthy cells and matrix with diseased ones. This approach, in fact, is already studied in other areas, such as oncology, where the importance of the tumor ECM in conditioning and promoting cancer cell behavior is well known [137,138].

### Translational Clinic Approaches

Cell-based therapy has been, by far, the most promising approach to treat SKM injuries in pre-clinical settings. Existing limitations of cell therapy approaches include issues related to autologous harvesting, expansion and sorting protocols, optimal dosage, and viability after transplantation. Recently, innovative approaches have been proposed to further enhance the efficacy of cell-based therapy, including improving biomaterial design to deliver and retain viable cells near the site of injury in a minimally invasive manner and modifying physical and chemical properties of biomaterials. Moreover, the multicellular anisotropic structure of SKM is more complex compared to other tissues and additive manufacturing techniques such as 3D bioprinting can naturally be applied to create functional tissues for translation into clinic. With this prominent aim, hurdles have been encountered in patient customization and scale up to produce the required quantity and tissue dimension [139].

To address the point on patient customization, Kang et al. developed a novel 3D bioprinting technique to design patient-specific constructs. They collected data via computer tomography and magnetic resonance imaging and incorporated into the 3D bioprinting software to produce human-scale muscle tissue, thus tailoring the construct design to the specific form of injured muscle in single patients [140]. Additive manufacturing acquires relevance also in advanced 3D in vitro drug testing approaches.

Mohammad et al. filled the gap between the lack of easy-to-use platforms, which are simple to fabricate and yield arrays of human muscle micro-tissues (hMMTs) in culture, and the reproducible physiological responses that can be assayed non-invasively. They designed and produced a 96-well culture platform for the bulk production of hMMTs using a combination of 3D printing and microfabrication techniques and demonstrated the feasibility of their system for phenotypic compound drug testing using an image based readout in a moderate throughput fashion [141].

## 8. Conclusions

We have observed that, although muscle biology has been a branch of medicine studied for a long time, and despite a lot of important mechanisms being known and considered fundamental in order to recreate in vitro a construct as similar as possible to the tissue in vivo, the exact combination of all chemical, biochemical, physical, and mechanical aspects as well as cellular ones is yet to be found. It is certainly important to start from a material that reflects as much as possible the complexity of SKM and that is tunable, in order to allow its manipulation with the addition of essential bioactive and structural factors for cellular behavior conditioning. Decellularized ECM biomaterial is a good compromise between tissue specificity and ease of handling, that permits cell engraftment, maturation but also deformation to induce myotubes alignment and orientation, all mandatory features necessary to obtain a functional muscle tissue.

**Figure 2:**
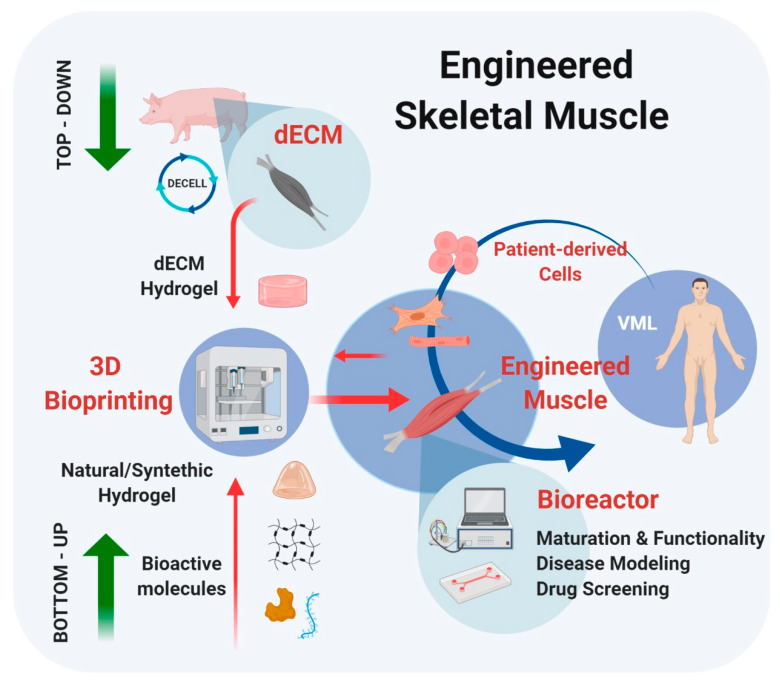
SKM tissue engineering strategies based on bottom-up and top-down parallel approaches. Bottom-up: generation of artificial biomaterials starting from natural-derived/synthetic hydrogels combined with specific bioactive molecules. Top-down: generation of dECM bioinks starting from animal (or human) samples. The 3D printing of these different types of SKM hydrogels will allow the production of (patient) specific tissue-like constructs for in vitro disease modeling and drug screening, and in vivo tissue replacement. Schematic was made using BioRender (https://app.biorender.com).

The innovative prospect of being able to use dECM hydrogel formulation as a bioink for 3D printing has certainly given a significant boost to the research in the field of myopathies or muscle reconstructions. The engineered constructs obtained with this technique, in fact, will allow studying in a fine and controlled way the arrangement of the structures, but also cells, peptides and small molecules positioning, favoring the study of drug treatment rather than the development of implantable muscle substitutes. In order to carry out a top-down approach, starting from human or animal biopsies, it will be possible to obtain dECM bioinks to be used with 3D printing. Further studies must be conducted on standardizing the techniques for obtaining dECM, of its transformation into bioink through different gelation methods, and on the printability of these inks to allow adequate deposition and structures formation. At the same time, a bottom-up approach using natural-derived/synthetic hydrogels could be performed, to obtain specific and peculiar printable materials to generate 3D constructs. All these approaches can flow into a parallel stimulation protocol to obtain a mature and functioning SKM construct (Figure 2) with the clinical perspective of being used in vitro in drug screening or directly in vivo as biological muscle implants.

## Figures and Tables

**Table 1:** Main growth factors involved in myogenesis. HGF: Hepatocyte growth factor; IGF-1: Insulin-like growth factor-1; FGF: Fibroblast growth factor; PDGF: Platelet-derived growth factor; VEGF: Vascular endothelial growth factor; TGF-β: Transforming growth factor beta.

Growth Factors	Role
HGH	Enhances satellite cells activation upon injury, tendency to delay differentiation in favor of proliferation [24].
IGF-1	Involved in all phases of myogenesis, promotes both satellite cells proliferation and differentiation [25,26].
FGF	Promotes satellite cells activation and proliferation, often in cooperation with HGH [21].
PDGF	Promotes myogenic proliferation and angiogenesis, delays terminal differentiation [21].
VEGF	Promotes vascularization, in association with satellite cells self-renewal [27].
TGF-β	Promotes fibroblast growth and collagen production, increases myoblast proliferation but is a potent inhibitor of muscle differentiation [28].

## References

[1] Frontera W.R., Ochala J. (2015). Skeletal Muscle: A Brief Review of Structure and Function. Behav. Genet..

[2] Gillies A.R., Lieber R.L. (2011). Structure and function of the skeletal muscle extracellular matrix. Muscle Nerve.

[3] Kjær M. (2004). Role of Extracellular Matrix in Adaptation of Tendon and Skeletal Muscle to Mechanical Loading. Physiol. Rev..

[4] Purslow P.P. (2002). The structure and functional significance of variations in the connective tissue within muscle. Comp. Biochem. Physiol. Part A Mol. Integr. Physiol..

[5] Gattazzo F., Urciuolo A., Bonaldo P. (2014). Extracellular matrix: A dynamic microenvironment for stem cell niche. Biochim. Biophys. Acta Gen. Subj..

[6] Mauro A. (1961). Satellite cell of skeletal muscle fibers. J. Biophys. Biochem. Cytol..

[7] Jana S., Levengood S.K.L., Zhang M. (2016). Anisotropic Materials for Skeletal-Muscle-Tissue Engineering. Adv. Mater..

[8] Dennis R.G., Smith B., Philp A., Donnelly K., Baar K. (2009). Bioreactors for guiding muscle tissue growth and development. Adv. Biochem. Eng. Biotechnol..

[9] Frantz C., Stewart K.M., Weaver V.M. (2010). The extracellular matrix at a glance. J. Cell Sci..

[10] Grzelkowska-Kowalczyk K. (2016). The Importance of Extracellular Matrix in Skeletal Muscle Development and Function. Composition and Function of the Extracellular Matrix in the Human Body.

[11] Thorsteinsdottir S., Deries M., Cachaço A.S., Bajanca F. (2011). The extracellular matrix dimension of skeletal muscle development. Dev. Biol..

[12] Janson I.A., Putnam A.J. (2015). Extracellular matrix elasticity and topography: Material-based cues that affect cell function via conserved mechanisms. J. Biomed. Mater. Res. Part A.

[13] Yue B. (2014). Biology of the extracellular matrix: An overview. J. Glaucoma.

[14] Sawicka K.M., Seeliger M., Musaev T., Macri L.K., Clark R.A.F. (2015). Fibronectin Interaction and Enhancement of Growth Factors: Importance for Wound Healing. Adv. Wound Care.

[15] Rayagiri S.S., Ranaldi D., Raven A., Mohamad Azhar N.I.F., Lefebvre O., Zammit P.S., Borycki A.G. (2018). Basal lamina remodeling at the skeletal muscle stem cell niche mediates stem cell self-renewal. Nat. Commun..

[16] Badylak S.F. (2004). Xenogeneic extracellular matrix as a scaffold for tissue reconstruction. Transpl. Immunol..

[17] Fischer M., Rikeit P., Knaus P., Coirault C. (2016). YAP-mediated mechanotransduction in skeletal muscle. Front. Physiol..

[18] Ramirez F., Rifkin D.B. (2003). Cell signaling events: A view from the matrix. Matrix Biol..

[19] Hynes R.O., Naba A. (2012). Overview of the matrisome—An inventory of extracellular matrix constituents and functions. Cold Spring Harb. Perspect. Biol..

[20] Dhawan J., Rando T.A. (2005). Stem cells in postnatal myogenesis: Molecular mechanisms of satellite cell quiescence, activation and replenishment. Trends Cell Biol..

[21] Syverud B.C., VanDusen K.W., Larkin L.M. (2016). Growth factors for skeletal muscle tissue engineering. Cells Tissues Org..

[22] Duan C., Ren H., Gao S. (2010). Insulin-like growth factors (IGFs), IGF receptors, and IGF-binding proteins: Roles in skeletal muscle growth and differentiation. Gen. Comp. Endocrinol..

[23] Lee P.H.U., Vandenburgh H.H. (2013). Skeletal muscle atrophy in bioengineered skeletal muscle: A new model system. Tissue Eng. Part A.

[24] Suzuki S., Yamanouchi K., Soeta C., Katakai Y., Harada R., Naito K., Tojo H. (2002). Skeletal muscle injury induces hepatocyte growth factor expression in spleen. Biochem. Biophys. Res. Commun..

[25] Chakravarthy M.V., Abraha T.W., Schwartz R.J., Fiorotto M.L., Booth F.W. (2000). Insulin-like growth factor-I extends in vitro replicative life span of skeletal muscle satellite cells by enhancing G1/S cell cycle progression via the activation of phosphatidylinositol 3’-kinase/Akt signaling pathway. J. Biol. Chem..

[26] Allen R.E., Boxhorn L.K. (1989). Regulation of skeletal muscle satellite cell proliferation and differentiation by transforming growth factor-beta, insulin-like growth factor I, and fibroblast growth factor. J. Cell. Physiol..

[27] Verma M., Asakura Y., Murakonda B.S.R., Pengo T., Latroche C., Chazaud B., McLoon L.K., Asakura A. (2018). Muscle Satellite Cell Cross-Talk with a Vascular Niche Maintains Quiescence via VEGF and Notch Signaling. Cell Stem Cell.

[28] Delaney K., Kasprzycka P., Ciemerych M.A., Zimowska M. (2017). The role of TGF-β1 during skeletal muscle regeneration. Cell Biol. Int..

[29] McCuller C., Callahan A.L. (2019). Physiology, Skeletal Muscle.

[30] Cheng C.S., Davis B.N.J., Madden L., Bursac N., Truskey G.A. (2014). Physiology and metabolism of tissue-engineered skeletal muscle. Exp. Biol. Med..

[31] Csapo R., Gumpenberger M., Wessner B. (2020). Skeletal Muscle Extracellular Matrix—What Do We Know About Its Composition, Regulation, and Physiological Roles? A Narrative Review. Front. Physiol..

[32] Ceafalan L.C., Popescu B.O., Hinescu M.E. (2014). Cellular players in skeletal muscle regeneration. Biomed. Res. Int..

[33] Ranjbar K., Fayazi B. (2020). Vascularisation of Skeletal Muscle. Muscle Cells—Recent Advances and Future Perspectives.

[34] Grounds M.D. (2008). Complexity of Extracellular Matrix and Skeletal Muscle Regeneration. Skeletal Muscle Repair and Regeneration.

[35] Schwander M., Shirasaki R., Pfaff S.L., Müller U. (2004). β1 integrins in muscle, but not in motor neurons, are required for skeletal muscle innervation. J. Neurosci..

[36] Kim J.H., Kim I., Seol Y.J., Ko I.K., Yoo J.J., Atala A., Lee S.J. (2020). Neural cell integration into 3D bioprinted skeletal muscle constructs accelerates restoration of muscle function. Nat. Commun..

[37] Mei Liu H. (1992). The role of extracellular matrix in peripheral nerve regeneration: A wound chamber study. Acta Neuropathol..

[38] Sugiura Y., Lin W. (2011). Neuron-glia interactions: The roles of Schwann cells in neuromuscular synapse formation and function. Biosci. Rep..

[39] Gehlert S., Jacko D. (2019). The role of the immune system in response to muscle damage. Dtsch. Z. Sportmed..

[40] Tidball J.G. (2005). Inflammatory processes in muscle injury and repair. Am. J. Physiol. Regul. Integr. Comp. Physiol..

[41] McKinnell I.W., Parise G., Rudnicki M.A. (2005). Muscle Stem Cells and Regenerative Myogenesis. Curr. Top. Dev. Biol..

[42] Cosgrove B.D., Sacco A., Gilbert P.M., Blau H.M. (2009). A home away from home: Challenges and opportunities in engineering in vitro muscle satellite cell niches. Differentiation.

[43] Chapman M.A., Meza R., Lieber R.L. (2016). Skeletal muscle fibroblasts in health and disease. Differentiation.

[44] Murphy M.M., Lawson J.A., Mathew S.J., Hutcheson D.A., Kardon G. (2011). Satellite cells, connective tissue fibroblasts and their interactions are crucial for muscle regeneration. Development.

[45] Urciuolo A., Urbani L., Perin S., Maghsoudlou P., Scottoni F., Gjinovci A., Collins-Hooper H., Loukogeorgakis S., Tyraskis A., Torelli S. (2018). Decellularised skeletal muscles allow functional muscle regeneration by promoting host cell migration. Sci. Rep..

[46] Salani S., Donadoni C., Rizzo F., Bresolin N., Comi G.P., Corti S. (2012). Generation of skeletal muscle cells from embryonic and induced pluripotent stem cells as an in vitro model and for therapy of muscular dystrophies. J. Cell. Mol. Med..

[47] Selvaraj S., Mondragon-Gonzalez R., Xu B., Magli A., Kim H., Lainé J., Kiley J., McKee H., Rinaldi F., Aho J. (2019). Screening identifies small molecules that enhance the maturation of human pluripotent stem cell-derived myotubes. eLife.

[48] Michela P., Chiara F., Martina P., Paolo D.C. (2014). Fetal stem cells and skeletal muscle regeneration: A therapeutic approach. Front. Aging Neurosci..

[49] Juhas M., Engelmayr G.C., Fontanella A.N., Palmer G.M., Bursac N. (2014). Biomimetic engineered muscle with capacity for vascular integration and functional maturation in vivo. Proc. Natl. Acad. Sci. USA.

[50] Trevisan C., Fallas M.E.A., Maghin E., Franzin C., Pavan P., Caccin P., Chiavegato A., Carraro E., Boso D., Boldrin F. (2019). Generation of a Functioning and Self-Renewing Diaphragmatic Muscle Construct. Stem Cells Transl. Med..

[51] Rao L., Qian Y., Khodabukus A., Ribar T., Bursac N. (2018). Engineering human pluripotent stem cells into a functional skeletal muscle tissue. Nat. Commun..

[52] Fuoco C., Sangalli E., Vono R., Testa S., Sacchetti B., Latronico M.V.G., Bernardini S., Madeddu P., Cesareni G., Seliktar D. (2014). 3D hydrogel environment rejuvenates aged pericytes for skeletal muscle tissue engineering. Front. Physiol..

[53] Maffioletti S.M., Sarcar S., Henderson A.B.H., Mannhardt I., Pinton L., Moyle L.A., Steele-Stallard H., Cappellari O., Wells K.E., Ferrari G. (2018). Three-Dimensional Human iPSC-Derived Artificial Skeletal Muscles Model Muscular Dystrophies and Enable Multilineage Tissue Engineering. Cell Rep..

[54] Peppas N.A., Merrill E.W. (1976). Poly(vinyl alcohol) hydrogels: Reinforcement of radiation-crosslinked networks by crystallization. J. Polym. Sci. Polym. Chem. Ed..

[55] Hill E., Boontheekul T., Mooney D.J. (2006). Designing scaffolds to enhance transplanted myoblast survival and migration. Tissue Eng..

[56] Ostrovidov S., Hosseini V., Ahadian S., Fujie T., Parthiban S.P., Ramalingam M., Bae H., Kaji H., Khademhosseini A. (2014). Skeletal muscle tissue engineering: Methods to form skeletal myotubes and their applications. Tissue Eng. Part B Rev..

[57] Qazi T.H., Mooney D.J., Pumberger M., Geißler S., Duda G.N. (2015). Biomaterials based strategies for skeletal muscle tissue engineering: Existing technologies and future trends. Biomaterials.

[58] Ji S., Guvendiren M. (2017). Recent Advances in Bioink Design for 3D Bioprinting of Tissues and Organs. Front. Bioeng. Biotechnol..

[59] Pollot B.E., Rathbone C.R., Wenke J.C., Guda T. (2018). Natural polymeric hydrogel evaluation for skeletal muscle tissue engineering. J. Biomed. Mater. Res. Part B Appl. Biomater..

[60] Matthias N., Hunt S.D., Wu J., Lo J., Smith Callahan L.A., Li Y., Huard J., Darabi R. (2018). Volumetric muscle loss injury repair using in situ fibrin gel cast seeded with muscle-derived stem cells (MDSCs). Stem Cell Res..

[61] Neal D., Sakar M.S., Ong L.L.S., Harry Asada H. (2014). Formation of elongated fascicle-inspired 3D tissues consisting of high-density, aligned cells using sacrificial outer molding. Lab Chip.

[62] Heher P., Maleiner B., Prüller J., Teuschl A.H., Kollmitzer J., Monforte X., Wolbank S., Redl H., Rünzler D., Fuchs C. (2015). A novel bioreactor for the generation of highly aligned 3D skeletal muscle-like constructs through orientation of fibrin via application of static strain. Acta Biomater..

[63] Chen P.Y., Yang K.C., Wu C.C., Yu J.H., Lin F.H., Sun J.S. (2014). Fabrication of large perfusable macroporous cell-laden hydrogel scaffolds using microbial transglutaminase. Acta Biomater..

[64] Paguirigan A.L., Beebe D.J. (2007). Protocol for the fabrication of enzymatically crosslinked gelatin microchannels for microfluidic cell culture. Nat. Protoc..

[65] Marcinczyk M., Elmashhady H., Talovic M., Dunn A., Bugis F., Garg K. (2017). Laminin-111 enriched fibrin hydrogels for skeletal muscle regeneration. Biomaterials.

[66] Visscher D.O., Farré-Guasch E., Helder M.N., Gibbs S., Forouzanfar T., van Zuijlen P.P., Wolff J. (2016). Advances in Bioprinting Technologies for Craniofacial Reconstruction. Trends Biotechnol..

[67] Williams J.M., Adewunmi A., Schek R.M., Flanagan C.L., Krebsbach P.H., Feinberg S.E., Hollister S.J., Das S. (2005). Bone tissue engineering using polycaprolactone scaffolds fabricated via selective laser sintering. Biomaterials.

[68] Guvendiren M., Burdick J.A. (2013). Engineering synthetic hydrogel microenvironments to instruct stem cells. Curr. Opin. Biotechnol..

[69] Xu Y., Li Z., Li X., Fan Z., Liu Z., Xie X., Guan J. (2015). Regulating myogenic differentiation of mesenchymal stem cells using thermosensitive hydrogels. Acta Biomater..

[70] Vannozzi L., Yasa I.C., Ceylan H., Menciassi A., Ricotti L., Sitti M. (2018). Self-Folded Hydrogel Tubes for Implantable Muscular Tissue Scaffolds. Macromol. Biosci..

[71] Browe D.P., Wood C., Sze M.T., White K.A., Scott T., Olabisi R.M., Freeman J.W. (2017). Characterization and optimization of actuating poly(ethylene glycol) diacrylate/acrylic acid hydrogels as artificial muscles. Polymer.

[72] Rich M.H., Lee M.K., Marshall N., Clay N., Chen J., Mahmassani Z., Boppart M., Kong H. (2015). Water-Hydrogel Binding Affinity Modulates Freeze-Drying-Induced Micropore Architecture and Skeletal Myotube Formation. Biomacromolecules.

[73] Hwang J.H., Kim I.G., Piao S., Jung A.R., Lee J.Y., Park K.D., Lee J.Y. (2013). Combination therapy of human adipose-derived stem cells and basic fibroblast growth factor hydrogel in muscle regeneration. Biomaterials.

[74] Mulyasasmita W., Cai L., Dewi R.E., Jha A., Ullmann S.D., Luong R.H., Huang N.F., Heilshorn S.C. (2014). Avidity-controlled hydrogels for injectable co-delivery of induced pluripotent stem cell-derived endothelial cells and growth factors. J. Control. Release.

[75] Pati F., Jang J., Ha D.H., Won Kim S., Rhie J.W., Shim J.H., Kim D.H., Cho D.W. (2014). Printing three-dimensional tissue analogues with decellularized extracellular matrix bioink. Nat. Commun..

[76] Piccoli M., Trevisan C., Maghin E., Franzin C., Pozzobon M. (2018). Mouse skeletal muscle decellularization. Methods Mol. Biol..

[77] Hoshiba T., Lu H., Kawazoe N., Chen G. (2010). Decellularized matrices for tissue engineering. Expert Opin. Biol. Ther..

[78] Brightman A.O., Rajwa B.P., Sturgis J.E., McCallister M.E., Robinson J.P., Voytik-Harbin S.L. (2000). Time-lapse confocal reflection microscopy of collagen fibrillogenesis and extracellular matrix assembly in vitro. Biopolymers.

[79] Voytik-Harbin S.L., Brightman A.O., Waisner B.Z., Robinson J.P., Lamar C.H. (1998). Small intestinal submucosa: A tissue-derived extracellular matrix that promotes tissue-specific growth and differentiation of cells in vitro. Tissue Eng..

[80] Hulmes D.J.S. (2008). Collagen diversity, synthesis and assembly. Collagen: Structure and Mechanics.

[81] Choi Y.J., Kim T.G., Jeong J., Yi H.G., Park J.W., Hwang W., Cho D.W. (2016). 3D Cell Printing of Functional Skeletal Muscle Constructs Using Skeletal Muscle-Derived Bioink. Adv. Healthc. Mater..

[82] Fu Y., Fan X., Tian C., Luo J., Zhang Y., Deng L., Qin T., Lv Q. (2016). Decellularization of porcine skeletal muscle extracellular matrix for the formulation of a matrix hydrogel: A preliminary study. J. Cell. Mol. Med..

[83] DeQuach J.A., Lin J.E., Cam C., Hu D., Salvatore M.A., Sheikh F., Christman K.L. (2012). Injectable skeletal muscle matrix hydrogel promotes neovascularization and muscle cell infiltration in a hindlimb ischemia model. Eur. Cells Mater..

[84] Ungerleider J.L., Johnson T.D., Rao N., Christman K.L. (2015). Fabrication and characterization of injectable hydrogels derived from decellularized skeletal and cardiac muscle. Methods.

[85] Fernández-Pérez J., Ahearne M. (2019). The impact of decellularization methods on extracellular matrix derived hydrogels. Sci. Rep..

[86] Choi Y.-J., Jun Y.-J., Kim D.Y., Yi H.-G., Chae S.-H., Kang J., Lee J., Gao G., Kong J.-S., Jang J. (2019). A 3D cell printed muscle construct with tissue-derived bioink for the treatment of volumetric muscle loss. Biomaterials.

[87] Kim W.J., Lee H., Lee J.U., Atala A., Yoo J.J., Lee S.J., Kim G.H. (2020). Efficient myotube formation in 3D bioprinted tissue construct by biochemical and topographical cues. Biomaterials.

[88] Yan W., George S., Fotadar U., Tyhovych N., Kamer A., Yost M.J., Price R.L., Haggart C.R., Holmes J.W., Terracio L. (2007). Tissue engineering of skeletal muscle. Tissue Eng..

[89] Nakayama K.H., Shayan M., Huang N.F. (2019). Engineering Biomimetic Materials for Skeletal Muscle Repair and Regeneration. Adv. Healthc. Mater..

[90] Akter F. (2016). Principles of Tissue Engineering. Tissue Engineering Made Easy.

[91] Grasman J.M., Do D.M., Page R.L., Pins G.D. (2015). Rapid release of growth factors regenerates force output in volumetric muscle loss injuries. Biomaterials.

[92] Liu G., Pareta R.A., Wu R., Shi Y., Zhou X., Liu H., Deng C., Sun X., Atala A., Opara E.C. (2013). Skeletal myogenic differentiation of urine-derived stem cells and angiogenesis using microbeads loaded with growth factors. Biomaterials.

[93] Ansari S., Chen C., Xu X., Annabi N., Zadeh H.H., Wu B.M., Khademhosseini A., Shi S., Moshaverinia A. (2016). Muscle Tissue Engineering Using Gingival Mesenchymal Stem Cells Encapsulated in Alginate Hydrogels Containing Multiple Growth Factors. Ann. Biomed. Eng..

[94] Bleiziffer O., Eriksson E., Yao F., Horch R.E., Kneser U. (2007). Gene transfer strategies in tissue engineering: Tissue Engineering Review Series. J. Cell. Mol. Med..

[95] Wang P., Berry D., Moran A., He F., Tam T., Chen L., Chen S. (2019). Controlled Growth Factor Release in 3D-Printed Hydrogels. Adv. Healthc. Mater..

[96] Han W.M., Mohiuddin M., Anderson S.E., García A.J., Jang Y.C. (2019). Wnt7a-releasing synthetic hydrogel enhances local skeletal muscle regeneration and muscle stem cell engraftment. bioRxiv.

[97] Huang K., Ozpinar E.W., Su T., Tang J., Shen D., Qiao L., Hu S., Li Z., Liang H., Mathews K. (2020). An off-the-shelf artificial cardiac patch improves cardiac repair after myocardial infarction in rats and pigs. Sci. Transl. Med..

[98] Stilhano R.S., Madrigal J.L., Wong K., Williams P.A., Martin P.K.M., Yamaguchi F.S.M., Samoto V.Y., Han S.W., Silva E.A. (2016). Injectable alginate hydrogel for enhanced spatiotemporal control of lentivector delivery in murine skeletal muscle. J. Control. Release.

[99] Hynes R.O. (2009). The extracellular matrix: Not just pretty fibrils. Science.

[100] Huang G., Li F., Zhao X., Ma Y., Li Y., Lin M., Jin G., Lu T.J., Genin G.M., Xu F. (2017). Functional and Biomimetic Materials for Engineering of the Three-Dimensional Cell Microenvironment. Chem. Rev..

[101] Li Y., Huang G., Zhang X., Wang L., Du Y., Lu T.J., Xu F. (2014). Engineering cell alignment in vitro. Biotechnol. Adv..

[102] Jiwlawat N., Lynch E.M., Napiwocki B.N., Stempien A., Ashton R.S., Kamp T.J., Crone W.C., Suzuki M. (2019). Micropatterned substrates with physiological stiffness promote cell maturation and Pompe disease phenotype in human induced pluripotent stem cell-derived skeletal myocytes. Biotechnol. Bioeng..

[103] Yang G.H., Jeon H., Kim G. (2017). Alternately plasma-roughened nanosurface of a hybrid scaffold for aligning myoblasts. Biofabrication.

[104] Tang S.W., Yuen W., Kaur I., Pang S.W., Voelcker N.H., Lam Y.W. (2020). Capturing instructive cues of tissue microenvironment by silica bioreplication. Acta Biomater..

[105] Rizzi R., Bearzi C., Mauretti A., Bernardini S., Cannata S., Gargioli C. (2012). Tissue engineering for skeletal muscle regeneration. Muscles. Ligaments Tendons J..

[106] Powell C.A., Smiley B.L., Mills J., Vandenburgh H.H. (2002). Mechanical stimulation improves tissue-engineered human skeletal muscle. Am. J. Physiol. Cell Physiol..

[107] Gilbert P.M., Havenstrite K.L., Magnusson K.E.G., Sacco A., Leonardi N.A., Kraft P., Nguyen N.K., Thrun S., Lutolf M.P., Blau H.M. (2010). Substrate elasticity regulates skeletal muscle stem cell self-renewal in culture. Science.

[108] Meyers M., Chawla K. (2008). Mechanical Behavior of Materials.

[109] Fedorovich N.E., Alblas J., Hennink W.E., Öner F.C., Dhert W.J.A. (2011). Organ printing: The future of bone regeneration?. Trends Biotechnol..

[110] Peltola S.M., Melchels F.P.W., Grijpma D.W., Kellomäki M. (2008). A review of rapid prototyping techniques for tissue engineering purposes. Ann. Med..

[111] Ouyang L., Yao R., Zhao Y., Sun W. (2016). Effect of bioink properties on printability and cell viability for 3D bioplotting of embryonic stem cells. Biofabrication.

[112] Groll J., Burdick J.A., Cho D.W., Derby B., Gelinsky M., Heilshorn S.C., Jüngst T., Malda J., Mironov V.A., Nakayama K. (2019). A definition of bioinks and their distinction from biomaterial inks. Biofabrication.

[113] Williams D., Thayer P., Martinez H., Gatenholm E., Khademhosseini A. (2018). A perspective on the physical, mechanical and biological specifications of bioinks and the development of functional tissues in 3D bioprinting. Bioprinting.

[114] Billiet T., Vandenhaute M., Schelfhout J., Van Vlierberghe S., Dubruel P. (2012). A review of trends and limitations in hydrogel-rapid prototyping for tissue engineering. Biomaterials.

[115] Chimene D., Lennox K.K., Kaunas R.R., Gaharwar A.K. (2016). Advanced Bioinks for 3D Printing: A Materials Science Perspective. Ann. Biomed. Eng..

[116] Guilak F., Cohen D.M., Estes B.T., Gimble J.M., Liedtke W., Chen C.S. (2009). Control of Stem Cell Fate by Physical Interactions with the Extracellular Matrix. Cell Stem Cell.

[117] Hoch E., Hirth T., Tovar G.E.M., Borchers K. (2013). Chemical tailoring of gelatin to adjust its chemical and physical properties for functional bioprinting. J. Mater. Chem. B.

[118] Ferris C.J., Gilmore K.J., Beirne S., McCallum D., Wallace G.G., In Het Panhuis M. (2013). Bio-ink for on-demand printing of living cells. Biomater. Sci..

[119] Blose K.J., Krawiec J.T., Weinbaum J.S., Vorp D.A. (2014). Bioreactors for tissue engineering purposes. Regenerative Medicine Applications in Organ Transplantation.

[120] Wang J., Khodabukus A., Rao L., Vandusen K., Abutaleb N., Bursac N. (2019). Engineered skeletal muscles for disease modeling and drug discovery. Biomaterials.

[121] Maleiner B., Tomasch J., Heher P., Spadiut O., Rünzler D., Fuchs C. (2018). The importance of biophysical and biochemical stimuli in dynamic skeletal muscle models. Front. Physiol..

[122] Kim H., Kim M.C., Asada H.H. (2019). Extracellular matrix remodelling induced by alternating electrical and mechanical stimulations increases the contraction of engineered skeletal muscle tissues. Sci. Rep..

[123] Vandenburgh H.H. (1982). Dynamic mechanical orientation of skeletal myofibers in vitro. Dev. Biol..

[124] Vandenburgh H.H. (1988). A computerized mechanical cell stimulator for tissue culture: Effects on skeletal muscle organogenesis. Vitr. Cell. Dev. Biol..

[125] Hatfaludy S., Shansky J., Vandenburgh H.H. (1989). Metabolic alterations induced in cultured skeletal muscle by stretch-relaxation activity. Am. J. Physiol. Cell Physiol..

[126] Moon D.G., Christ G., Stitzel J.D., Atala A., Yoo J.J. (2008). Cyclic mechanical preconditioning improves engineered muscle contraction. Tissue Eng. Part A.

[127] Juhas M., Ye J., Bursac N. (2016). Design, evaluation, and application of engineered skeletal muscle. Methods.

[128] Goldspink G., Scutt A., Loughna P.T., Wells D.J., Jaenicke T., Gerlach G.F. (1992). Gene expression in skeletal muscle in response to stretch and force generation. Am. J. Physiol..

[129] Khodabukus A., Baar K. (2012). Defined electrical stimulation emphasizing excitability for the development and testing of engineered skeletal muscle. Tissue Eng. Part C Methods.

[130] Brevet A., Pinto E., Peacock J., Stockdale F.E. (1976). Myosin synthesis increased by electrical stimulation of skeletal muscle cell cultures. Science.

[131] Khodabukus A., Baar K. (2016). Factors that affect tissue-engineered skeletal muscle function and physiology. Cells Tissues Organs.

[132] Benam K.H., Dauth S., Hassell B., Herland A., Jain A., Jang K.-J., Karalis K., Kim H.J., MacQueen L., Mahmoodian R. (2015). Engineered In Vitro Disease Models. Annu. Rev. Pathol. Mech. Dis..

[133] Bersini S., Arrigoni C., Lopa S., Bongio M., Martin I., Moretti M. (2016). Engineered miniaturized models of musculoskeletal diseases. Drug Discov. Today.

[134] Serena E., Zatti S., Reghelin E., Pasut A., Cimetta E., Elvassore N. (2010). Soft substrates drive optimal differentiation of human healthy and dystrophic myotubes. Integr. Biol..

[135] Vandenburgh H., Shansky J., Benesch-Lee F., Skelly K., Spinazzola J.M., Saponjian Y., Tseng B.S. (2009). Automated drug screening with contractile muscle tissue engineered from dystrophic myoblasts. FASEB J..

[136] Vandenburgh H., Shansky J., Benesch-Lee F., Barbata V., Reid J., Thorrez L., Valentini R., Crawford G. (2008). Drug-screening platform based on the contractility of tissue-engineered muscle. Muscle Nerve.

[137] Tian X., Werner M.E., Roche K.C., Hanson A.D., Foote H.P., Yu S.K., Warner S.B., Copp J.A., Lara H., Wauthier E.L. (2018). Organ-specific metastases obtained by culturing colorectal cancer cells on tissue-specific decellularized scaffolds. Nat. Biomed. Eng..

[138] D’Angelo E., Natarajan D., Sensi F., Ajayi O., Fassan M., Mammano E., Pilati P., Pavan P., Bresolin S., Preziosi M. (2020). Patient-Derived Scaffolds of Colorectal Cancer Metastases as an Organotypic 3D Model of the Liver Metastatic Microenvironment. Cancers.

[139] Qazi T.H., Duda G.N., Ort M.J., Perka C., Geissler S., Winkler T. (2019). Cell therapy to improve regeneration of skeletal muscle injuries. J. Cachexia. Sarcopenia Muscle.

[140] Kang H.W., Lee S.J., Ko I.K., Kengla C., Yoo J.J., Atala A. (2016). A 3D bioprinting system to produce human-scale tissue constructs with structural integrity. Nat. Biotechnol..

[141] Afshar M.E., Abraha H.Y., Bakooshli M.A., Davoudi S., Thavandiran N., Tung K., Ahn H., Ginsberg H.J., Zandstra P.W., Gilbert P.M. (2020). A 96-well culture platform enables longitudinal analyses of engineered human skeletal muscle microtissue strength. Sci. Rep..

